# Methyl 3-[4-(4-nitro­benz­yloxy)phen­yl]propano­ate

**DOI:** 10.1107/S1600536812024701

**Published:** 2012-06-02

**Authors:** Linden Servinis, Bronwyn L. Fox, Peter C. Healy, Luke C. Henderson

**Affiliations:** a Institute for Frontier Materials, SRC for Biotechnology, Chemistry and Systems Biology, Faculty of Science and Technology, Deakin University, Victoria 3216, Australia; b Queensland Micro and Nanotechnology Centre, Griffith University, Brisbane 4111, Australia

## Abstract

The title compound, C_17_H_17_NO_5_, crystallizes with two mol­ecules (*A* and *B*) in the asymmetric unit. The conformational structures of the two mol­ecules show small but significant differences in the dihedral angles between the two aryl rings with values of 18.8 (1)° for mol­ecule *A* and 7.5 (1)° for mol­ecule *B*. In mol­ecule *A*, the propano­ate group is twisted out of the plane of the benzene group [C_ar_—C_ar_—C—C torsion angle = −44.9 (2)°], while for mol­ecule *B*, this group lies closer to the plane [C_ar_—C_ar_—C—C torsion angle = 8.6 (3)°]. C—H⋯O inter­actions characterize the crystal-packing inter­actions in this compound.

## Related literature

For background to the functionalization of carbon nanostructures and fibres, see: Forohar *et al.* (2011 ▶); Moradi *et al.* (2012 ▶); Nierengarten *et al.* (2004 ▶). For the synthesis, see: Greene *et al.* (1999 ▶). For related structures, see: Li & Chen (2008 ▶); Wang *et al.* (2007 ▶).
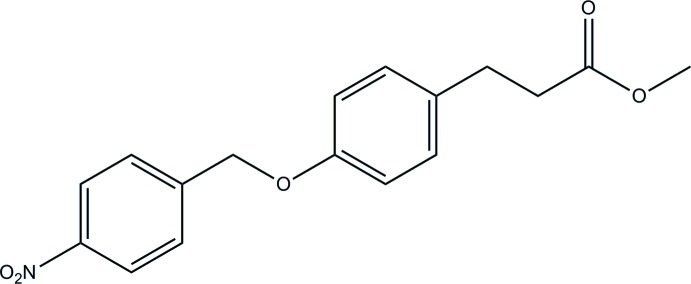


## Experimental

### 

#### Crystal data

C_17_H_17_NO_5_
*M* *_r_* = 315.32
Triclinic, 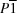

*a* = 10.7434 (5) Å
*b* = 10.9408 (5) Å
*c* = 14.7225 (6) Å
α = 100.085 (4)°
β = 102.451 (4)°
γ = 110.329 (4)°
*V* = 1524.34 (14) Å^3^
*Z* = 4
Mo *K*α radiation
μ = 0.10 mm^−1^
*T* = 223 K
0.48 × 0.30 × 0.27 mm

#### Data collection

Oxford Diffraction Gemini S Ultra diffractometer
Absorption correction: multi-scan (*CrysAlis PRO*; Agilent, 2012 ▶) *T*_min_ = 0.953, *T*_max_ = 0.973
10836 measured reflections
5352 independent reflections
4439 reflections with *I* > 2σ(*I*)
*R*_int_ = 0.023

#### Refinement

*R*[*F* ^2^ > 2σ(*F* ^2^)] = 0.044
*wR*(*F* ^2^) = 0.113
*S* = 1.02
5352 reflections
415 parameters
H-atom parameters constrained
Δρ_max_ = 0.30 e Å^−3^
Δρ_min_ = −0.21 e Å^−3^

### 

Data collection: *CrysAlis PRO* (Agilent, 2012 ▶); cell refinement: *CrysAlis PRO*; data reduction: *CrysAlis PRO*; program(s) used to solve structure: *TEXSAN* (Molecular Structure Corporation, 2001 ▶) and *SIR97* (Altomare *et al.*, 1999 ▶); program(s) used to refine structure: *TEXSAN* and *SHELXL97* (Sheldrick, 2008 ▶); molecular graphics: *ORTEP-3 for Windows* (Farrugia, 1997 ▶); software used to prepare material for publication: *PLATON* (Spek, 2009 ▶) and *publCIF* (Westrip, 2010 ▶).

## Supplementary Material

Crystal structure: contains datablock(s) global, I. DOI: 10.1107/S1600536812024701/tk5106sup1.cif

Structure factors: contains datablock(s) I. DOI: 10.1107/S1600536812024701/tk5106Isup2.hkl

Supplementary material file. DOI: 10.1107/S1600536812024701/tk5106Isup3.cml

Additional supplementary materials:  crystallographic information; 3D view; checkCIF report

## Figures and Tables

**Table 1:** Hydrogen-bond geometry (Å, °)

*D*—H⋯*A*	*D*—H	H⋯*A*	*D*⋯*A*	*D*—H⋯*A*
C3—H3⋯O3	0.95	2.43	2.757 (2)	100
C6—H6⋯O6^i^	0.95	2.53	3.362 (2)	146
C19—H19⋯O2^ii^	0.95	2.55	3.430 (2)	153
C20—H20⋯O8	0.95	2.40	2.736 (2)	101
C7—H72⋯O9	0.95	2.49	3.336 (3)	149

Symmetry codes: (i) 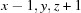; (ii) 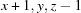.

**Fig. 1.**
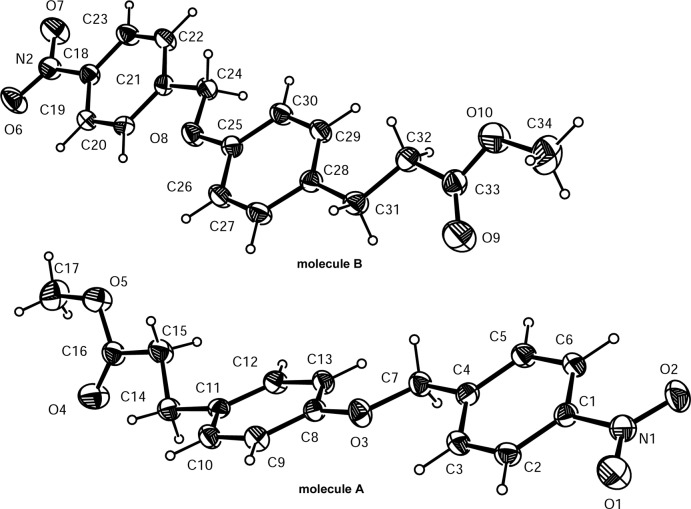
View of molecules A and B of the title compound with the atom numbering scheme. Displacement ellipsoids for non-H atoms are drawn at the 40% probability level. Hydrogen atoms are shown as spheres of arbitrary radius.

**Fig. 2.**
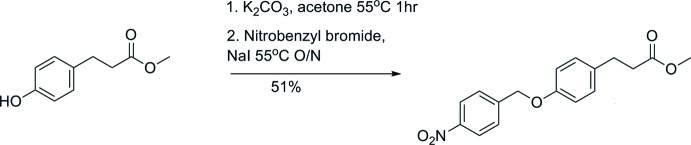
Reaction scheme for the preparation of the title compound.

## supplementary crystallographic information

### Comment

The structure of the title compound 1 was determined as part of an ongoing project investigating the surface functionalization of carbon nanostructures and carbon fibers, which have massive application in chemistry and materials science, respectively (Forohar *et al.*, 2011; Moradi *et al.*, 2012; Nierengarten *et al.*, 2004). The oxidized nitro group present on the aryl ring serves as an excellent diagnostic handle for surface characterization using X-Ray Photoelectron Spectroscopy (XPS). This compound provides a synthetically versatile scaffold with an alkyl carboxyl group which can be used for compound derivatization and surface attachment strategies. Additionally the 4-nitrophenyl group serves as an excellent protecting group for alcohols, including phenols, which can readily be removed by hydrogenolysis or electrolytically (Greene *et al.*, 1999).

The compound crystallizes with two independent molecules in the asymmetric unit (Fig. 1). The bond lengths and angles for each molecule are in accord with related structures (*e.g.* Li & Chen, 2008; Wang *et al.*, 2007). The conformational structure of the two molecules show small but significant differences in the dihedral angles between the two aryl rings with values of 18.8 (1)° for molecule A and 7.5 (1)° for molecule B. In molecule A, the propanoate group is twisted out of the plane of the phenyl group with the C12—C11—C14—C15 torsion angle = -44.9 (2)°, while for molecule B, this group lies closer to the plane with the torsion angle C29—C30—C31—C32 = 8.6 (3)°. C—H···O interactions characterize the crystal packing interactions in this compound (Table 1).

### Experimental

To a solution of (CH_3_)_2_CO (10 ml) and methyl 4-hydroxyphenylpropanoate (0.194 g, 1.28 mmol), was added K_2_CO_3_ (0.195 g, 1.41 mmol) followed by reflux at 55 °C for 1 h. Nitrobenzyl bromide (0.305 g, 1.41 mmol) and NaI (0.192 g, 1.28 mmol) were added to the solution and the reaction stirred a further 15 h at 55 °C (Fig. 2). The resulting crude mixture was filtered, and the residual precipitate washed with acetone and diethyl ether. The organic phases were combined, dried over MgSO_4_, and solvent removed *in vacuo*. Purification by column chromatography (9:1, PET ether:ethyl acetate) gave a colourless crystalline solid was confirmed to be the desired propanoate 1 (51%, 0.206 g). The purified solid was dissolved in toluene and slowly evaporated over 3 days to give well formed single crystals suitable for X-ray diffraction studies. ν_(max)_ cm ^-1^: 2922 (aromatic C—H), 2830 (methyl C—H, aliphatic –CH_2_–), 1728 (ester C=O), 1511 (aromatic C—C=C), 1160 (ether C—O—C). ^1^H NMR (270 MHz, CDCl_3_): *δ*= 8.23 (d, 2H, ^3^*J*_HH_= 8.64 Hz, Ar*H*), 7.59 (d, 2H, ^3^*J*_HH_ = 8.37 Hz, Ar*H*), 7.12 (d, 2H, ^3^*J*_HH_ = 8.64 Hz, Ar*H*), 6.87 (d, 2H, ^3^*J*_HH_ = 8.91 Hz, Ar*H*), 5.14 (s, 2H, C*H*_2_Bn), 3.65 (s, 3H, C*H*_3_), 2.89 (t, 2H, ^3^*J*_HH_ = 7.29, 8.10 Hz, C*H*_2_), 2.59 (t, 2H, ^3^*J*_HH_ = 7.83 Hz, C*H*_2_). ^13^C NMR (400 MHz, CDCl_3_): *δ* = 173.41, 156.74, 147.65, 144.75, 133.69, 129.51, 127.66, 123.91, 114.95, 68.81, 51.69, 35.95, 30.15. M.P. 110.5–116.8 °C. MS, *m/z* found: MNa^+^ 338.09985, (C_17_H_17_NO_5_), MNa^+^ requires 338.09989.

### Refinement

The carbon-bound H atoms were constrained as riding atoms with C—H = 0.95 Å. *U*_iso_(H) values were set at 1.2*U*_eq_ of the parent atom.

### Crystal data

**Table:**

C_17_H_17_NO_5_	*Z* = 4
*M**_r_* = 315.32	*F*(000) = 664
Triclinic, *P*1	*D*_x_ = 1.374 Mg m^−^^3^
Hall symbol: -P 1	Mo *K*α radiation, λ = 0.71070 Å
*a* = 10.7434 (5) Å	Cell parameters from 3807 reflections
*b* = 10.9408 (5) Å	θ = 3.3–32.3°
*c* = 14.7225 (6) Å	µ = 0.10 mm^−^^1^
α = 100.085 (4)°	*T* = 223 K
β = 102.451 (4)°	Block, colourless
γ = 110.329 (4)°	0.48 × 0.30 × 0.27 mm
*V* = 1524.34 (14) Å^3^	

### Data collection

**Table:**

Oxford Diffraction Gemini S Ultra diffractometer	5352 independent reflections
Radiation source: Enhance (Mo) X-ray Source	4439 reflections with *I* > 2σ(*I*)
Graphite monochromator	*R*_int_ = 0.023
Detector resolution: 16.0774 pixels mm^-1^	θ_max_ = 25.0°, θ_min_ = 3.3°
ω and φ scans	*h* = −12→12
Absorption correction: multi-scan (*CrysAlis PRO*; Agilent, 2012)	*k* = −13→12
*T*_min_ = 0.953, *T*_max_ = 0.973	*l* = −16→17
10836 measured reflections	

### Refinement

**Table:**

Refinement on *F*^2^	Primary atom site location: structure-invariant direct methods
Least-squares matrix: full	Secondary atom site location: difference Fourier map
*R*[*F*^2^ > 2σ(*F*^2^)] = 0.044	Hydrogen site location: inferred from neighbouring sites
*wR*(*F*^2^) = 0.113	H-atom parameters constrained
*S* = 1.02	*w* = 1/[σ^2^(*F*_o_^2^) + (0.053*P*)^2^ + 0.4295*P*] where *P* = (*F*_o_^2^ + 2*F*_c_^2^)/3
5352 reflections	(Δ/σ)_max_ < 0.001
415 parameters	Δρ_max_ = 0.30 e Å^−^^3^
0 restraints	Δρ_min_ = −0.21 e Å^−^^3^

### Special details

**Table:**

Geometry. Bond distances, angles *etc*. have been calculated using the rounded fractional coordinates. All su's are estimated from the variances of the (full) variance-covariance matrix. The cell e.s.d.'s are taken into account in the estimation of distances, angles and torsion angles
Refinement. Refinement on *F*^2^ for ALL reflections except those flagged by the user for potential systematic errors. Weighted *R*-factors *wR* and all goodnesses of fit *S* are based on *F*^2^, conventional *R*-factors *R* are based on *F*, with *F* set to zero for negative *F*^2^. The observed criterion of *F*^2^ > σ(*F*^2^) is used only for calculating -*R*-factor-obs *etc*. and is not relevant to the choice of reflections for refinement. *R*-factors based on *F*^2^ are statistically about twice as large as those based on *F*, and *R*-factors based on ALL data will be even larger.

### Fractional atomic coordinates and isotropic or equivalent isotropic displacement parameters (Å^2^)

**Table:**

	*x*	*y*	*z*	*U*_iso_*/*U*_eq_	
O1	0.38540 (15)	0.44558 (14)	0.75138 (10)	0.0536 (5)	
O2	0.46341 (14)	0.66419 (13)	0.79376 (9)	0.0442 (4)	
O3	0.77377 (12)	0.46142 (12)	0.43690 (8)	0.0361 (4)	
O4	1.27684 (16)	0.40392 (16)	0.06737 (10)	0.0584 (6)	
O5	1.20730 (15)	0.54474 (14)	−0.00107 (10)	0.0527 (5)	
N1	0.45760 (15)	0.55726 (15)	0.74593 (10)	0.0353 (5)	
C1	0.54163 (16)	0.56360 (17)	0.67913 (11)	0.0296 (5)	
C2	0.54634 (17)	0.44504 (17)	0.63167 (12)	0.0327 (5)	
C3	0.63065 (17)	0.45237 (17)	0.57180 (12)	0.0328 (5)	
C4	0.70756 (16)	0.57620 (17)	0.55938 (11)	0.0295 (5)	
C5	0.69733 (17)	0.69303 (17)	0.60625 (12)	0.0324 (5)	
C6	0.61555 (17)	0.68788 (17)	0.66726 (11)	0.0320 (5)	
C7	0.80603 (17)	0.59037 (17)	0.49921 (11)	0.0327 (5)	
C8	0.85805 (16)	0.45517 (17)	0.37967 (11)	0.0295 (5)	
C9	0.82338 (17)	0.32775 (17)	0.31975 (12)	0.0339 (5)	
C10	0.89918 (18)	0.31117 (17)	0.25746 (12)	0.0337 (5)	
C11	1.01274 (17)	0.41883 (17)	0.25341 (11)	0.0298 (5)	
C12	1.04699 (17)	0.54498 (17)	0.31516 (12)	0.0334 (5)	
C13	0.97100 (17)	0.56459 (17)	0.37775 (11)	0.0327 (5)	
C14	1.09481 (18)	0.39482 (18)	0.18584 (12)	0.0358 (6)	
C15	1.1326 (2)	0.49946 (19)	0.13152 (13)	0.0433 (6)	
C16	1.21374 (19)	0.47485 (18)	0.06437 (12)	0.0383 (6)	
C17	1.2821 (2)	0.5335 (2)	−0.07054 (15)	0.0580 (8)	
O6	1.58768 (17)	0.98754 (15)	−0.31164 (11)	0.0605 (6)	
O7	1.61594 (17)	1.19282 (15)	−0.30738 (11)	0.0609 (6)	
O8	1.21905 (13)	0.96352 (12)	0.01260 (8)	0.0394 (4)	
O9	0.8214 (2)	0.88318 (16)	0.45479 (12)	0.0749 (7)	
O10	0.76481 (17)	1.05413 (17)	0.43287 (11)	0.0653 (6)	
N2	1.57398 (17)	1.09104 (16)	−0.27962 (11)	0.0419 (5)	
C18	1.50153 (17)	1.09276 (17)	−0.20589 (12)	0.0332 (5)	
C19	1.46203 (18)	0.98197 (17)	−0.16998 (12)	0.0331 (5)	
C20	1.38825 (17)	0.98116 (17)	−0.10369 (11)	0.0325 (5)	
C21	1.35429 (17)	1.09054 (16)	−0.07405 (11)	0.0310 (5)	
C22	1.39757 (19)	1.20163 (17)	−0.11081 (13)	0.0386 (6)	
C23	1.4716 (2)	1.20386 (18)	−0.17701 (13)	0.0398 (6)	
C24	1.27354 (18)	1.09383 (17)	−0.00279 (12)	0.0348 (5)	
C25	1.14952 (17)	0.95195 (17)	0.08110 (11)	0.0324 (5)	
C26	1.1068 (2)	0.82765 (18)	0.10146 (13)	0.0396 (6)	
C27	1.0374 (2)	0.80774 (18)	0.17001 (13)	0.0393 (6)	
C28	1.00838 (17)	0.90992 (17)	0.22033 (11)	0.0323 (5)	
C29	1.05156 (18)	1.03236 (17)	0.19785 (12)	0.0356 (5)	
C30	1.12121 (19)	1.05475 (17)	0.12914 (12)	0.0362 (5)	
C31	0.93478 (19)	0.88386 (18)	0.29658 (13)	0.0386 (6)	
C32	0.89227 (19)	0.99506 (18)	0.33696 (12)	0.0375 (6)	
C33	0.82259 (19)	0.96733 (19)	0.41304 (13)	0.0396 (6)	
C34	0.6981 (3)	1.0422 (3)	0.50823 (17)	0.0715 (10)	
H2	0.49290	0.36040	0.64000	0.0390*	
H3	0.63580	0.37210	0.53910	0.0390*	
H5	0.74740	0.77740	0.59610	0.0390*	
H6	0.61030	0.76790	0.70020	0.0380*	
H9	0.74740	0.25190	0.32160	0.0410*	
H10	0.87310	0.22360	0.21610	0.0400*	
H12	1.12480	0.62020	0.31470	0.0400*	
H13	0.99630	0.65220	0.41880	0.0390*	
H71	0.89890	0.62510	0.54050	0.0390*	
H72	0.79690	0.65080	0.46160	0.0390*	
H141	1.04080	0.30910	0.14010	0.0430*	
H151	1.18690	0.58520	0.17720	0.0520*	
H152	1.04910	0.50070	0.09450	0.0520*	
H171	1.27440	0.59180	−0.11030	0.0700*	
H172	1.24360	0.44260	−0.10960	0.0700*	
H173	1.37730	0.55870	−0.03740	0.0700*	
H412	1.17840	0.39380	0.22290	0.0430*	
H19	1.48510	0.90740	−0.19030	0.0400*	
H20	1.36040	0.90540	−0.07820	0.0390*	
H22	1.37600	1.27700	−0.09020	0.0460*	
H23	1.50130	1.27990	−0.20200	0.0480*	
H26	1.12520	0.75620	0.06840	0.0470*	
H27	1.00860	0.72200	0.18330	0.0470*	
H29	1.03290	1.10390	0.23060	0.0430*	
H30	1.14920	1.14010	0.11530	0.0430*	
H241	1.33320	1.15820	0.05670	0.0420*	
H242	1.19920	1.11860	−0.02750	0.0420*	
H311	0.85330	0.80260	0.26850	0.0460*	
H312	0.99550	0.87260	0.34870	0.0460*	
H321	0.97320	1.07690	0.36420	0.0450*	
H322	0.82990	1.00540	0.28530	0.0450*	
H341	0.61190	0.96540	0.48450	0.0860*	
H342	0.75650	1.03200	0.56210	0.0860*	
H343	0.68190	1.12150	0.52750	0.0860*	

### Atomic displacement parameters (Å^2^)

**Table:**

	*U*^11^	*U*^22^	*U*^33^	*U*^12^	*U*^13^	*U*^23^
O1	0.0659 (9)	0.0450 (8)	0.0700 (9)	0.0238 (7)	0.0479 (8)	0.0268 (7)
O2	0.0553 (8)	0.0453 (8)	0.0450 (7)	0.0283 (7)	0.0286 (6)	0.0099 (6)
O3	0.0381 (7)	0.0342 (7)	0.0408 (6)	0.0145 (5)	0.0230 (5)	0.0077 (5)
O4	0.0766 (10)	0.0764 (10)	0.0645 (9)	0.0567 (9)	0.0462 (8)	0.0363 (8)
O5	0.0738 (10)	0.0639 (9)	0.0571 (8)	0.0446 (8)	0.0477 (8)	0.0353 (7)
N1	0.0377 (8)	0.0406 (9)	0.0376 (8)	0.0207 (7)	0.0189 (6)	0.0149 (7)
C1	0.0313 (8)	0.0366 (9)	0.0281 (8)	0.0181 (7)	0.0128 (7)	0.0118 (7)
C2	0.0371 (9)	0.0301 (9)	0.0371 (9)	0.0154 (8)	0.0159 (7)	0.0139 (7)
C3	0.0392 (9)	0.0321 (9)	0.0346 (9)	0.0200 (8)	0.0157 (7)	0.0095 (7)
C4	0.0300 (8)	0.0352 (9)	0.0280 (8)	0.0166 (7)	0.0105 (7)	0.0104 (7)
C5	0.0352 (9)	0.0300 (9)	0.0358 (9)	0.0134 (7)	0.0151 (7)	0.0119 (7)
C6	0.0382 (9)	0.0297 (9)	0.0333 (9)	0.0180 (8)	0.0140 (7)	0.0081 (7)
C7	0.0358 (9)	0.0323 (9)	0.0337 (9)	0.0153 (7)	0.0153 (7)	0.0086 (7)
C8	0.0316 (9)	0.0347 (9)	0.0295 (8)	0.0178 (7)	0.0135 (7)	0.0115 (7)
C9	0.0319 (9)	0.0313 (9)	0.0393 (9)	0.0116 (7)	0.0142 (7)	0.0096 (7)
C10	0.0382 (9)	0.0305 (9)	0.0360 (9)	0.0177 (8)	0.0136 (7)	0.0066 (7)
C11	0.0359 (9)	0.0347 (9)	0.0291 (8)	0.0216 (8)	0.0135 (7)	0.0132 (7)
C12	0.0350 (9)	0.0337 (9)	0.0382 (9)	0.0148 (8)	0.0185 (7)	0.0148 (8)
C13	0.0383 (9)	0.0295 (9)	0.0340 (9)	0.0154 (8)	0.0157 (7)	0.0080 (7)
C14	0.0431 (10)	0.0412 (10)	0.0360 (9)	0.0257 (8)	0.0194 (8)	0.0142 (8)
C15	0.0600 (12)	0.0428 (11)	0.0473 (10)	0.0307 (10)	0.0337 (9)	0.0186 (9)
C16	0.0467 (11)	0.0380 (10)	0.0400 (10)	0.0215 (9)	0.0226 (8)	0.0129 (8)
C17	0.0643 (14)	0.0803 (16)	0.0516 (12)	0.0355 (13)	0.0408 (11)	0.0289 (11)
O6	0.0928 (12)	0.0517 (9)	0.0691 (9)	0.0448 (9)	0.0535 (9)	0.0250 (7)
O7	0.0843 (11)	0.0526 (9)	0.0737 (10)	0.0331 (8)	0.0540 (9)	0.0354 (8)
O8	0.0530 (8)	0.0326 (7)	0.0440 (7)	0.0208 (6)	0.0280 (6)	0.0134 (5)
O9	0.1260 (15)	0.0617 (10)	0.0817 (11)	0.0514 (10)	0.0743 (11)	0.0456 (9)
O10	0.0896 (12)	0.0874 (12)	0.0684 (9)	0.0624 (10)	0.0579 (9)	0.0423 (9)
N2	0.0499 (9)	0.0414 (9)	0.0444 (8)	0.0220 (8)	0.0238 (7)	0.0165 (7)
C18	0.0350 (9)	0.0328 (9)	0.0337 (9)	0.0139 (8)	0.0135 (7)	0.0094 (7)
C19	0.0377 (9)	0.0305 (9)	0.0337 (9)	0.0173 (8)	0.0110 (7)	0.0072 (7)
C20	0.0375 (9)	0.0291 (9)	0.0315 (8)	0.0138 (7)	0.0094 (7)	0.0100 (7)
C21	0.0320 (9)	0.0302 (9)	0.0280 (8)	0.0123 (7)	0.0065 (7)	0.0049 (7)
C22	0.0476 (11)	0.0305 (9)	0.0449 (10)	0.0205 (8)	0.0198 (8)	0.0103 (8)
C23	0.0497 (11)	0.0310 (9)	0.0469 (10)	0.0180 (8)	0.0222 (9)	0.0174 (8)
C24	0.0412 (10)	0.0304 (9)	0.0347 (9)	0.0157 (8)	0.0138 (8)	0.0077 (7)
C25	0.0354 (9)	0.0345 (9)	0.0320 (8)	0.0172 (8)	0.0134 (7)	0.0099 (7)
C26	0.0532 (11)	0.0318 (10)	0.0450 (10)	0.0240 (9)	0.0235 (9)	0.0119 (8)
C27	0.0493 (11)	0.0305 (9)	0.0470 (10)	0.0197 (8)	0.0211 (9)	0.0163 (8)
C28	0.0329 (9)	0.0335 (9)	0.0328 (9)	0.0148 (7)	0.0109 (7)	0.0105 (7)
C29	0.0445 (10)	0.0312 (9)	0.0387 (9)	0.0203 (8)	0.0188 (8)	0.0096 (7)
C30	0.0459 (10)	0.0297 (9)	0.0406 (9)	0.0182 (8)	0.0195 (8)	0.0137 (7)
C31	0.0449 (10)	0.0393 (10)	0.0409 (10)	0.0207 (9)	0.0196 (8)	0.0169 (8)
C32	0.0423 (10)	0.0378 (10)	0.0387 (9)	0.0170 (8)	0.0189 (8)	0.0150 (8)
C33	0.0456 (11)	0.0375 (10)	0.0380 (9)	0.0147 (9)	0.0188 (8)	0.0119 (8)
C34	0.0716 (16)	0.108 (2)	0.0606 (14)	0.0485 (15)	0.0448 (13)	0.0278 (14)

### Geometric parameters (Å, º)

**Table:**

O1—N1	1.226 (2)	C13—H13	0.9500
O2—N1	1.229 (2)	C14—H412	0.9500
O3—C7	1.422 (2)	C14—H141	0.9500
O3—C8	1.373 (2)	C15—H151	0.9500
O4—C16	1.195 (3)	C15—H152	0.9500
O5—C16	1.336 (2)	C17—H172	0.9500
O5—C17	1.444 (3)	C17—H173	0.9500
O6—N2	1.219 (2)	C17—H171	0.9500
O7—N2	1.225 (2)	C18—C19	1.376 (3)
O8—C24	1.420 (2)	C18—C23	1.383 (3)
O8—C25	1.377 (2)	C19—C20	1.383 (3)
O9—C33	1.191 (3)	C20—C21	1.395 (3)
O10—C33	1.328 (3)	C21—C22	1.390 (2)
O10—C34	1.446 (3)	C21—C24	1.502 (3)
N1—C1	1.466 (2)	C22—C23	1.383 (3)
N2—C18	1.467 (3)	C25—C26	1.385 (3)
C1—C2	1.385 (3)	C25—C30	1.382 (3)
C1—C6	1.379 (3)	C26—C27	1.382 (3)
C2—C3	1.387 (3)	C27—C28	1.396 (3)
C3—C4	1.388 (3)	C28—C29	1.382 (3)
C4—C5	1.394 (3)	C28—C31	1.518 (3)
C4—C7	1.505 (3)	C29—C30	1.389 (3)
C5—C6	1.380 (3)	C31—C32	1.514 (3)
C8—C13	1.387 (3)	C32—C33	1.493 (3)
C8—C9	1.388 (2)	C19—H19	0.9500
C9—C10	1.380 (3)	C20—H20	0.9500
C10—C11	1.392 (3)	C22—H22	0.9500
C11—C14	1.511 (3)	C23—H23	0.9500
C11—C12	1.389 (2)	C24—H241	0.9500
C12—C13	1.392 (3)	C24—H242	0.9500
C14—C15	1.501 (3)	C26—H26	0.9500
C15—C16	1.500 (3)	C27—H27	0.9500
C2—H2	0.9500	C29—H29	0.9500
C3—H3	0.9500	C30—H30	0.9500
C5—H5	0.9500	C31—H311	0.9500
C6—H6	0.9500	C31—H312	0.9500
C7—H71	0.9500	C32—H321	0.9500
C7—H72	0.9500	C32—H322	0.9500
C9—H9	0.9500	C34—H341	0.9500
C10—H10	0.9500	C34—H342	0.9500
C12—H12	0.9500	C34—H343	0.9500
			
O1···C17^i^	3.185 (3)	C29···H242^x^	2.9700
O1···C23^ii^	3.350 (3)	C30···H242	2.7400
O2···C9^iii^	3.212 (2)	C30···H241	2.7000
O2···C17^i^	3.252 (3)	C32···H29	2.5600
O3···C1^iii^	3.342 (2)	H2···O1	2.4300
O3···N1^iii^	3.1678 (19)	H2···O7^ii^	2.7300
O4···C24^iv^	3.354 (3)	H3···O3	2.4300
O6···C6^v^	3.362 (2)	H5···H72	2.4800
O6···C34^v^	3.196 (3)	H5···O9	2.6900
O7···C34^v^	3.368 (3)	H6···O2	2.4300
O7···C2^vi^	3.315 (2)	H6···O6^i^	2.5300
O9···C7	3.336 (3)	H9···C19^viii^	2.9000
O1···H172^i^	2.8000	H9···H19^viii^	2.5900
O1···H2	2.4300	H10···H20^viii^	2.6000
O1···H23^ii^	2.6300	H10···H29^iv^	2.4900
O2···H19^i^	2.5500	H10···H141	2.3300
O2···H241^vii^	2.6200	H10···C20^viii^	2.8200
O2···H6	2.4300	H12···H151	2.2800
O2···H171^i^	2.6900	H12···O7^ix^	2.8600
O3···H3	2.4300	H12···C15	2.8200
O4···H173	2.6000	H13···H72	2.3600
O4···H412	2.7200	H13···C7	2.5300
O4···H172	2.6800	H13···H71	2.2700
O4···H141	2.9000	H19···O6	2.4300
O5···H152^viii^	2.6300	H19···O2^v^	2.5500
O5···H26	2.8600	H19···H9^viii^	2.5900
O6···H6^v^	2.5300	H20···O8	2.4000
O6···H19	2.4300	H20···C20^ix^	3.0600
O6···H342^v^	2.8500	H20···H10^viii^	2.6000
O7···H23	2.4400	H22···H242	2.5500
O7···H151^ix^	2.6700	H23···O1^vi^	2.6300
O7···H343^v^	2.7400	H23···O7	2.4400
O7···H12^ix^	2.8600	H26···O5	2.8600
O7···H2^vi^	2.7300	H27···C12	3.0400
O7···H341^x^	2.9200	H27···H311	2.5800
O8···H20	2.4000	H27···H172^viii^	2.5300
O9···H5	2.6900	H29···H321	2.2300
O9···H311	2.8600	H29···H322	2.4500
O9···H312	2.7000	H29···C32	2.5600
O9···H72	2.4900	H29···H10^xii^	2.4900
O9···H342	2.4500	H29···C10^xii^	3.0900
O9···H341	2.7900	H30···C14^xii^	3.0900
N1···C8^iii^	3.439 (2)	H30···C24	2.5000
N1···O3^iii^	3.1678 (19)	H30···H242	2.2700
C1···O3^iii^	3.342 (2)	H30···H241	2.2800
C2···C5^iii^	3.585 (2)	H30···H141^xii^	2.5200
C2···O7^ii^	3.315 (2)	H71···C8^xi^	3.1000
C2···C4^iii^	3.381 (2)	H71···C9^xi^	3.0500
C3···C12^xi^	3.492 (3)	H71···H13	2.2700
C3···C5^iii^	3.533 (3)	H71···C13	2.7200
C4···C2^iii^	3.381 (2)	H71···C10^xi^	3.0800
C4···C11^xi^	3.591 (2)	H72···O9	2.4900
C5···C29^vii^	3.550 (2)	H72···C13	2.7800
C5···C3^iii^	3.533 (3)	H72···H5	2.4800
C5···C2^iii^	3.585 (2)	H72···H13	2.3600
C6···C30^vii^	3.573 (2)	H141···H10	2.3300
C6···O6^i^	3.362 (2)	H141···O4	2.9000
C7···O9	3.336 (3)	H141···H30^iv^	2.5200
C8···N1^iii^	3.439 (2)	H151···C12	2.7800
C9···O2^iii^	3.212 (2)	H151···H12	2.2800
C10···C20^viii^	3.532 (3)	H151···O7^ix^	2.6700
C11···C4^xi^	3.591 (2)	H152···O5^viii^	2.6300
C12···C3^xi^	3.492 (3)	H171···O2^v^	2.6900
C17···O1^v^	3.185 (3)	H172···O4	2.6800
C17···O2^v^	3.252 (3)	H172···O1^v^	2.8000
C20···C10^viii^	3.532 (3)	H172···H27^viii^	2.5300
C20···C20^ix^	3.312 (2)	H173···O4	2.6000
C23···O1^vi^	3.350 (3)	H173···C22^ix^	2.9700
C24···O4^xii^	3.354 (3)	H241···C30	2.7000
C29···C5^vii^	3.550 (2)	H241···H30	2.2800
C30···C6^vii^	3.573 (2)	H241···O2^vii^	2.6200
C34···O6^i^	3.196 (3)	H242···C30	2.7400
C34···O7^i^	3.368 (3)	H242···H30	2.2700
C1···H412^xi^	2.8700	H242···C28^x^	3.1000
C2···H412^xi^	2.9600	H242···C29^x^	2.9700
C3···H412^xi^	3.0500	H242···H22	2.5500
C4···H412^xi^	3.0900	H311···O9	2.8600
C5···H412^xi^	3.0500	H311···H27	2.5800
C6···H412^xi^	2.9400	H312···O9	2.7000
C7···H13	2.5300	H312···H342^vii^	2.4500
C8···H71^xi^	3.1000	H321···C29	2.7700
C9···H71^xi^	3.0500	H321···H29	2.2300
C10···H71^xi^	3.0800	H322···C29	2.9000
C10···H29^iv^	3.0900	H322···H29	2.4500
C12···H27	3.0400	H322···C21^x^	3.0700
C12···H151	2.7800	H322···C22^x^	3.0200
C13···H72	2.7800	H341···O9	2.7900
C13···H71	2.7200	H341···O7^x^	2.9200
C14···H30^iv^	3.0900	H342···H312^vii^	2.4500
C15···H12	2.8200	H342···O6^i^	2.8500
C19···H9^viii^	2.9000	H342···O9	2.4500
C20···H20^ix^	3.0600	H343···O7^i^	2.7400
C20···H10^viii^	2.8200	H412···C3^xi^	3.0500
C21···H322^x^	3.0700	H412···C6^xi^	2.9400
C22···H322^x^	3.0200	H412···C4^xi^	3.0900
C22···H173^ix^	2.9700	H412···C5^xi^	3.0500
C24···H30	2.5000	H412···O4	2.7200
C28···H242^x^	3.1000	H412···C1^xi^	2.8700
C29···H322	2.9000	H412···C2^xi^	2.9600
C29···H321	2.7700		
			
C7—O3—C8	117.05 (14)	H172—C17—H173	110.00
C16—O5—C17	117.38 (17)	O5—C17—H171	109.00
C24—O8—C25	116.46 (14)	O5—C17—H172	109.00
C33—O10—C34	116.9 (2)	O5—C17—H173	109.00
O2—N1—C1	118.30 (15)	H171—C17—H172	109.00
O1—N1—O2	123.36 (16)	N2—C18—C19	118.83 (17)
O1—N1—C1	118.34 (15)	N2—C18—C23	118.89 (16)
O6—N2—O7	122.95 (18)	C19—C18—C23	122.26 (18)
O6—N2—C18	118.45 (16)	C18—C19—C20	118.77 (17)
O7—N2—C18	118.59 (17)	C19—C20—C21	120.55 (16)
C2—C1—C6	122.26 (17)	C20—C21—C22	119.13 (17)
N1—C1—C2	118.87 (16)	C20—C21—C24	122.46 (15)
N1—C1—C6	118.86 (15)	C22—C21—C24	118.41 (16)
C1—C2—C3	118.64 (17)	C21—C22—C23	120.91 (18)
C2—C3—C4	120.39 (17)	C18—C23—C22	118.37 (17)
C5—C4—C7	118.21 (16)	O8—C24—C21	109.36 (15)
C3—C4—C5	119.39 (16)	O8—C25—C26	116.11 (16)
C3—C4—C7	122.37 (16)	O8—C25—C30	124.36 (16)
C4—C5—C6	120.96 (17)	C26—C25—C30	119.53 (17)
C1—C6—C5	118.32 (16)	C25—C26—C27	119.88 (18)
O3—C7—C4	109.47 (15)	C26—C27—C28	121.93 (18)
O3—C8—C9	115.45 (16)	C27—C28—C29	116.73 (17)
O3—C8—C13	125.10 (15)	C27—C28—C31	119.85 (16)
C9—C8—C13	119.45 (16)	C29—C28—C31	123.41 (16)
C8—C9—C10	120.04 (17)	C28—C29—C30	122.41 (17)
C9—C10—C11	121.99 (16)	C25—C30—C29	119.52 (17)
C10—C11—C12	116.98 (17)	C28—C31—C32	115.05 (16)
C10—C11—C14	120.00 (16)	C31—C32—C33	113.63 (16)
C12—C11—C14	123.00 (17)	O9—C33—O10	122.7 (2)
C11—C12—C13	122.09 (17)	O9—C33—C32	126.3 (2)
C8—C13—C12	119.43 (16)	O10—C33—C32	111.03 (17)
C11—C14—C15	114.51 (17)	C18—C19—H19	121.00
C14—C15—C16	114.25 (17)	C20—C19—H19	121.00
O4—C16—C15	126.18 (17)	C19—C20—H20	120.00
O4—C16—O5	123.33 (19)	C21—C20—H20	120.00
O5—C16—C15	110.49 (17)	C21—C22—H22	120.00
C1—C2—H2	121.00	C23—C22—H22	120.00
C3—C2—H2	121.00	C18—C23—H23	121.00
C4—C3—H3	120.00	C22—C23—H23	121.00
C2—C3—H3	120.00	O8—C24—H241	109.00
C6—C5—H5	120.00	O8—C24—H242	109.00
C4—C5—H5	120.00	C21—C24—H241	110.00
C1—C6—H6	121.00	C21—C24—H242	110.00
C5—C6—H6	121.00	H241—C24—H242	109.00
C4—C7—H72	109.00	C25—C26—H26	120.00
O3—C7—H71	109.00	C27—C26—H26	120.00
H71—C7—H72	109.00	C26—C27—H27	119.00
C4—C7—H71	109.00	C28—C27—H27	119.00
O3—C7—H72	109.00	C28—C29—H29	119.00
C10—C9—H9	120.00	C30—C29—H29	119.00
C8—C9—H9	120.00	C25—C30—H30	120.00
C11—C10—H10	119.00	C29—C30—H30	120.00
C9—C10—H10	119.00	C28—C31—H311	108.00
C11—C12—H12	119.00	C28—C31—H312	108.00
C13—C12—H12	119.00	C32—C31—H311	108.00
C12—C13—H13	120.00	C32—C31—H312	108.00
C8—C13—H13	120.00	H311—C31—H312	109.00
C11—C14—H141	108.00	C31—C32—H321	108.00
C11—C14—H412	108.00	C31—C32—H322	108.00
C15—C14—H141	108.00	C33—C32—H321	108.00
C15—C14—H412	108.00	C33—C32—H322	108.00
H141—C14—H412	109.00	H321—C32—H322	109.00
C16—C15—H151	108.00	O10—C34—H341	109.00
C16—C15—H152	108.00	O10—C34—H342	109.00
C14—C15—H152	108.00	O10—C34—H343	109.00
C14—C15—H151	108.00	H341—C34—H342	110.00
H151—C15—H152	109.00	H341—C34—H343	109.00
H171—C17—H173	110.00	H342—C34—H343	109.00
			
C8—O3—C7—C4	−177.23 (14)	C9—C10—C11—C12	−0.1 (3)
C7—O3—C8—C9	−179.95 (14)	C10—C11—C12—C13	−0.8 (3)
C7—O3—C8—C13	−0.4 (2)	C14—C11—C12—C13	−179.17 (17)
C17—O5—C16—O4	−0.6 (3)	C12—C11—C14—C15	−44.9 (2)
C17—O5—C16—C15	179.13 (16)	C10—C11—C14—C15	136.77 (18)
C24—O8—C25—C30	5.1 (2)	C11—C12—C13—C8	0.6 (3)
C25—O8—C24—C21	175.97 (14)	C11—C14—C15—C16	−179.85 (16)
C24—O8—C25—C26	−174.82 (16)	C14—C15—C16—O5	161.32 (16)
C34—O10—C33—O9	−0.4 (3)	C14—C15—C16—O4	−19.0 (3)
C34—O10—C33—C32	−177.68 (19)	N2—C18—C23—C22	177.03 (17)
O2—N1—C1—C6	−4.7 (2)	C19—C18—C23—C22	−1.0 (3)
O1—N1—C1—C2	−5.9 (2)	C23—C18—C19—C20	0.9 (3)
O1—N1—C1—C6	175.45 (16)	N2—C18—C19—C20	−177.13 (16)
O2—N1—C1—C2	173.96 (16)	C18—C19—C20—C21	0.2 (3)
O7—N2—C18—C19	−175.91 (18)	C19—C20—C21—C24	179.58 (17)
O6—N2—C18—C19	5.3 (3)	C19—C20—C21—C22	−1.1 (3)
O6—N2—C18—C23	−172.80 (18)	C20—C21—C22—C23	1.0 (3)
O7—N2—C18—C23	6.0 (3)	C22—C21—C24—O8	169.92 (16)
C6—C1—C2—C3	1.4 (3)	C24—C21—C22—C23	−179.66 (17)
N1—C1—C2—C3	−177.26 (16)	C20—C21—C24—O8	−10.7 (2)
N1—C1—C6—C5	178.22 (16)	C21—C22—C23—C18	0.1 (3)
C2—C1—C6—C5	−0.4 (3)	O8—C25—C26—C27	179.40 (17)
C1—C2—C3—C4	−0.5 (3)	C30—C25—C26—C27	−0.5 (3)
C2—C3—C4—C7	176.84 (16)	O8—C25—C30—C29	−179.27 (17)
C2—C3—C4—C5	−1.2 (3)	C26—C25—C30—C29	0.7 (3)
C3—C4—C5—C6	2.2 (3)	C25—C26—C27—C28	0.0 (3)
C5—C4—C7—O3	−163.63 (15)	C26—C27—C28—C31	−178.60 (18)
C7—C4—C5—C6	−175.92 (16)	C26—C27—C28—C29	0.5 (3)
C3—C4—C7—O3	18.3 (2)	C27—C28—C29—C30	−0.3 (3)
C4—C5—C6—C1	−1.4 (3)	C27—C28—C31—C32	−172.41 (17)
C13—C8—C9—C10	−1.4 (3)	C29—C28—C31—C32	8.6 (3)
O3—C8—C13—C12	−179.03 (16)	C31—C28—C29—C30	178.69 (18)
C9—C8—C13—C12	0.5 (3)	C28—C29—C30—C25	−0.2 (3)
O3—C8—C9—C10	178.24 (16)	C28—C31—C32—C33	−178.92 (16)
C8—C9—C10—C11	1.1 (3)	C31—C32—C33—O10	−168.35 (17)
C9—C10—C11—C14	178.37 (17)	C31—C32—C33—O9	14.5 (3)

Symmetry codes: (i) *x*−1, *y*, *z*+1; (ii) *x*−1, *y*−1, *z*+1; (iii) −*x*+1, −*y*+1, −*z*+1; (iv) *x*, *y*−1, *z*; (v) *x*+1, *y*, *z*−1; (vi) *x*+1, *y*+1, *z*−1; (vii) −*x*+2, −*y*+2, −*z*+1; (viii) −*x*+2, −*y*+1, −*z*; (ix) −*x*+3, −*y*+2, −*z*; (x) −*x*+2, −*y*+2, −*z*; (xi) −*x*+2, −*y*+1, −*z*+1; (xii) *x*, *y*+1, *z*.

### Hydrogen-bond geometry (Å, º)

**Table:**

*D*—H···*A*	*D*—H	H···*A*	*D*···*A*	*D*—H···*A*
C3—H3···O3	0.95	2.43	2.757 (2)	100
C6—H6···O6^i^	0.95	2.53	3.362 (2)	146
C19—H19···O2^v^	0.95	2.55	3.430 (2)	153
C20—H20···O8	0.95	2.40	2.736 (2)	101
C7—H72···O9	0.95	2.49	3.336 (3)	149

Symmetry codes: (i) *x*−1, *y*, *z*+1; (v) *x*+1, *y*, *z*−1.
